# Functional Analysis and Characterization of Differential Coexpression Networks

**DOI:** 10.1038/srep13295

**Published:** 2015-08-18

**Authors:** Chia-Lang Hsu, Hsueh-Fen Juan, Hsuan-Cheng Huang

**Affiliations:** 1 Department of Life Science, National Taiwan University, Taipei 10617, Taiwan; 2 Graduate Institute of Biomedical Electronics and Bioinformatics, National Taiwan University, Taipei 10617, Taiwan; 3 Institute of Molecular and Cellular Biology, National Taiwan University, Taipei 10617, Taiwan; 4 Institute of Biomedical Informatics, Center of Systems and Synthetic Biology, National Yang-Ming University, Taipei 11221, Taiwan

## Abstract

Differential coexpression analysis is emerging as a complement to conventional differential gene expression analysis. The identified differential coexpression links can be assembled into a differential coexpression network (DCEN) in response to environmental stresses or genetic changes. Differential coexpression analyses have been successfully used to identify condition-specific modules; however, the structural properties and biological significance of general DCENs have not been well investigated. Here, we analyzed two independent *Saccharomyces cerevisiae* DCENs constructed from large-scale time-course gene expression profiles in response to different situations. Topological analyses show that DCENs are tree-like networks possessing scale-free characteristics, but not small-world. Functional analyses indicate that differentially coexpressed gene pairs in DCEN tend to link different biological processes, achieving complementary or synergistic effects. Furthermore, the gene pairs lacking common transcription factors are sensitive to perturbation and hence lead to differential coexpression. Based on these observations, we integrated transcriptional regulatory information into DCEN and identified transcription factors that might cause differential coexpression by gain or loss of activation in response to different situations. Collectively, our results not only uncover the unique structural characteristics of DCEN but also provide new insights into interpretation of DCEN to reveal its biological significance and infer the underlying gene regulatory dynamics.

In biological systems, distinct groups of molecules that are functionally coordinated, physically interacting or co-regulated, drive complex biological processes. To dissect the complexity of biological systems, a complete map of intermolecular interactions is required. Therefore, numerous large networks have been measured systematically for humans and many other model species. These networks include physical attachments underlying protein-protein interactions, kinase-substrate interactions, protein-DNA interactions and metabolic reactions, as well as functional associations such as epistasis, synthetic lethality relationships and correlated expression between genes^1^,^2^. These various molecular networks have been successfully applied to address different biological questions, such as identification of disease genes^1^ and drug discovery^3^,^4^.

Molecular interactions can change dramatically in response to different conditions, such as environmental stresses and genetic changes. In other words, a molecule interaction can be present in some conditions, but absent in others. However, most large-scale networks to date have been measured under a single static condition, usually standard laboratory growth media. Understanding of network dynamics has been achieved to some extent by integrating static networks with gene expression profiles^5^. However, these approaches are typically unable to identify new interactions that are condition-specific. To completely understand the cellular dynamics, various differential network analyses have been proposed by experimental mapping of networks across multiple conditions^6^. Analogous to differential gene expression analyses, differential network analyses involve pairwise subtraction of interactions that have been mapped in differential experimental conditions. The extracted interactions are differentially present, absent or modified and relevant to the studied condition or phenotype. Several differential network mappings have revealed that the architecture of a molecular network can be massively re-wired during a cellular response and demonstrated the power of differential network analyses for elucidating biological mechanisms^7^,^8^.

Coexpression networks are typically constructed from gene expression data using correlation-based inference methods. These networks have been commonly used to reveal gene functions and investigate gene regulatory systems^9^,^10^. However, similar to other static molecular networks, gene coexpression networks only disclose the gene regulatory interactions under specific conditions. To understand the dynamics of cellular regulation, differential coexpression analysis incorporating regulatory changes between different conditions is emerging. Differential coexpression analysis investigates the differences among gene interconnections by calculating the expression correlation change of each gene pair between conditions. A large variety of differential coexpression analysis methods have been developed, such as the Log Ratio of Connections^11^, Average Specific Connection^12^, Weighted Gene Coexpression Network Analysis (WGCNA)^13^, Differential Coexpression profile (DCp)^14^, Differential Coexpression enrichment (DCe)^14^, Differential Correlation in Expression for meta-module Recovery (DICER)^15^, and DiffCoEx^16^. For a review on the methods for differential coexpression networking, see Ref. 17. Differential coexpression analysis has been applied to successfully identify the differential coexpression modules, a group of genes strongly correlated under one condition but not the other^17^,^18^,^19^,^20^, and differential expressed genes. Moreover, differential coexpression may indicate the rewiring of transcriptional networks in response to disease or adaption to different environments. In brief, differential coexpression network (DCEN) can provide a more informative picture of the dynamic changes in gene regulatory networks.

Previous studies with differential coexpression analyses mainly emphasized looking for differential expressed genes and differential coexpression modules rather than whole networks^17^,^18^,^19^,^20^. In this study, we investigated the structural characteristics and biological significance of DCEN and used time-course gene expression data to construct coexpression networks and obtain DCEN by comparing the networks between two biological conditions. Several network structural features were quantified to investigate the common and unique properties between DCEN and other differential networks. Then, we incorporated other information to interpret the biological significance of DCENs. Finally, we offered a computational method to identify differential activation of transcription factors inferred from DCENs.

## Results and Discussion

### Construction of differential coexpression networks

We constructed differential coexpression networks (DCENs) by using time-course gene expression data. Since biological systems are dynamic, temporal profiles of gene expression during a given biological process can often provide more insight about how genes are dependent on each other in a given biological process. To find the common properties of DCENs, we employed two distinct large-scale time-course gene expression datasets from Gene Expression Omnibus (GEO)^21^. The first dataset (Dataset 1, GEO accession GSE4158) was designed for understanding the dynamics of transcriptional response to changing environments by administering two different pulses (0.2 g/l and 2.0 g/l) of glucose on steady-state cultures of *Saccharomyces cerevisiae*^22^. The gene expression profiles over 12 (including 2, 4, 6, 8, 10, 15, 20, 30, 45, 90, 120, and 150 minutes) and 14 time points (including 3, 5, 7, 10, 15, 20, 30, 45, 90, 120, 150, 180, 210 and 240 minutes) were measured after addition of 0.2 g/l and 2.0 g/l glucose into cells, respectively. For the second data set (Dataset 2, GEO accession GSE3635 and GSE5283), the gene expression profiling of wild-type and strains with deleted YOX1 and YHP1 was performed to understand the regulation of transcription factor YOX1 and YHP1 during the cell cycle of *Saccharomyces cerevisiae*^23^. The wild-type and mutant cells were collected at 0, 10, 20, 30, 40, 50, 60, 70, 80, 90, 100, 110, and 120 minutes after synchronization with alpha factor.

**Figure 1:**
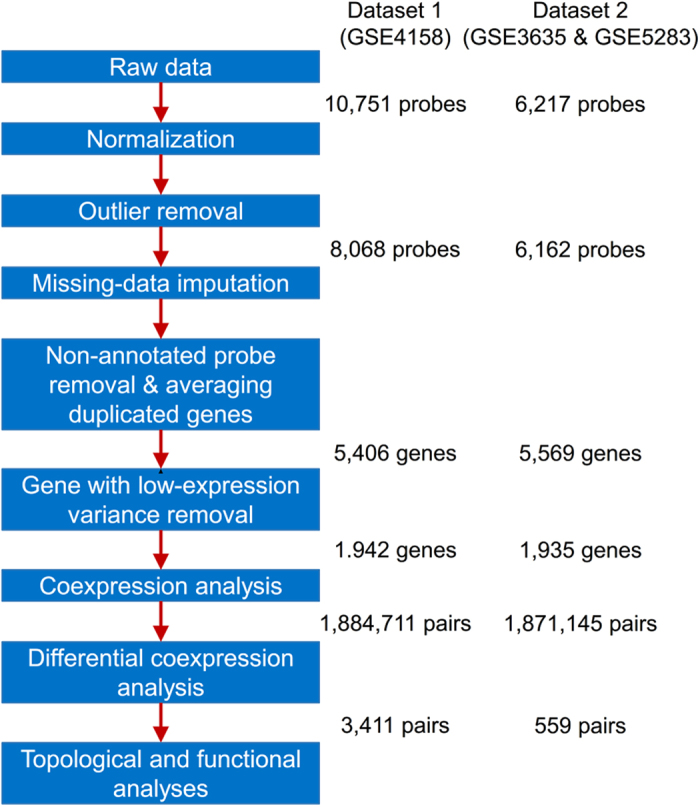
Overview of differential coexpression analysis pipeline. The order of analysis is shown for the data pre-processing and differential coexpression analysis. The values depicted to the right are the number of available data points remaining after a given procedure.

The gene expression data were pre-processed using the following steps: normalization, outlier removal, missing-data imputation, non-annotated probe removal and averaging duplicated genes, as well as removal of genes with low-expression variance (Fig. 1). These steps not only discarded non-informative genes but also reduced the computing time of coexpression analysis. Subsequently, we applied the method proposed by Wally *et al.*^20^ with slight modification to deduce differentially coexpressed links (DCELs), which were further assembled into DCENs. The abbreviations DCEN1 and DCEN2 were used to denote the DCEN constructed using Dataset 1 and Dataset 2, respectively. DCEN1 represents the coexpression changes of cells that were treated with low (0.2 g/l) to high (2.0 g/l) glucose concentration, and DCEN2 reveals coexpression changes in cells with and without YOX and YHP1 mutations. DCEN1 consisted of 1425 genes and 3411 DCELs with 1795 positive and 1616 negative links (Table S1), whereas DCEN2 consisted of 611 genes and 559 DCELs with 284 positive and 275 negative links (Table S2). These two DCENs were used for further analyses.

Additionally, gene pairs with highly correlated expression patterns (Spearman correlation coefficient >0.95) under both conditions were considered as constitutively coexpressed links (CCEL), and the network assembled from CCELs was termed the constitutive coexpression network (CCEN). The CCEN from Dataset 1 (CCEN1) contained 569 genes and 1545 links, and that from Dataset 2 (CCEN2) contained 262 genes and 570 links.

### Structural characteristics of differential coexpression networks

To unravel whether the structural properties of a DCEN are similar to those of other biological networks, the following quantities were measured in this study: average degree (*< k >*), maximum degree (Max. *k*), exponent of the degree distribution (γ), average clustering coefficient (*C*), diameter (*D*), and average shortest path length (*L*). To clarify whether the observed structural properties are unique for DCENs or common for differential networks, topological analysis on other types of differential networks is necessary. Therefore, we collected a differential genetic interaction network (DGIN) that arises when cells are challenged by DNA damage^8^ for comparison with the DCENs. Moreover, because the DCEN1 and DCEN2 could be broken down into 22 and 94 connected components, respectively (Figure S1), the corresponding largest components were used for the topology measurements.

**Figure 2:**
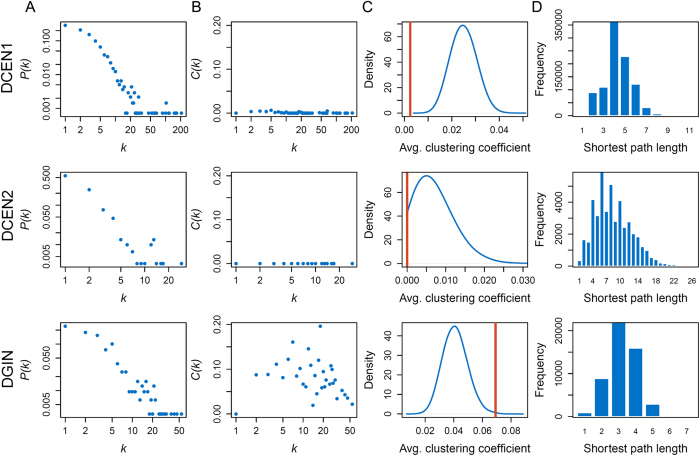
Topological properties of differential networks. (**A**) Degree distributions of differential networks. *P*(*k*) is the number of genes with *k* links. (**B**) Clustering coefficient versus degree for differential networks. *C(k)* denotes the average clustering coefficient of all genes with *k* links. (**C**) Distributions of average clustering coefficient for random networks (blue line). The red line denotes the average clustering coefficient of the given differential networks. (**D**) Distributions of shortest path lengths for differential networks.

The structural features of all differential networks are summarized in Table 1 and Fig. 2. Although the edge and node counts of DCEN1 are larger than those of DCEN2, the number of positive and negative differential links in each network is balanced. The degree distributions of differential networks suggest that they possess the scale-free properties^24^ with their exponent of degree distribution (γ) in the range between 2 and 4 (Fig. 2A and Table 1). The DGIN might have an intrinsic hierarchical structure as the average clustering coefficient of node decreases when the node degree increases^24^ (Fig. 2B). However, the average clustering coefficients of DCENs are quite low. To clarify whether the low average clustering coefficient is due to their specific degree distributions, we generated the background distribution of average clustering coefficients from 10^5^ degree-preserving random networks. Surprisingly, the average clustering coefficient of DCEN was significantly lower than the background distribution (*P*-value < 10^−4^ for both DCEN1 and DCEN2) in contrast to that of DGIN (Fig. 2C). Moreover, the average shortest path length of DCEN was greater than that of DGIN (Fig. 2D).

The comparison of these structural properties between differential networks indicates that DCENs differ substantially from the DGIN and other well-known biological networks, such as protein-protein interaction networks^24^. Although DCEN possesses the scale-free properties, DCEN is a tree-like network due to its low average clustering coefficient and high average shortest path length. These unique properties of DCEN might be inherited from those of coexpression networks. Coexpression network can be considered as a signed network that consists of positive (correlation) and negative (anti-correlation) links. Since correlations have transitive characteristics, two genes with common coexpressed and/or anti-coexpressed gene are expected to express simultaneously^25^. In terms of triad, which consists of three mutually linked nodes and is the smallest unit of a complete graph, a signed network generally contains four types of triads^25^. However, the transitive property of coexpression links indicates that only two types of triads can be observed in the coexpression network, that is, triads with an odd number of positive links^25^. Therefore, in a comparison between two coexpression networks, only two out of three links in a triad will reveal significant differences (Figure S2), and this property could result in the structure of DCEN as a tree-like network. To further confirm this characteristic of DCEN, we performed differential coexpression analysis on randomized gene expression data between conditions and counted the number of triads in the resultant random DCEN. Surprisingly, the proportion of observed triads in real DCEN is significantly lower than that in random DCEN (*P*-value < 10^−5^, Figure S3). We also found that strong coexpression triads tend to be coexpressed in another condition (Figure S4). That is, genes rarely form triads in DCENs. Additionally, we used a different method, DCe^14^, to identify differentially coexpressed links and re-analyze the topological properties of the constructed DCEN. As shown in Figure S5, the networks constructed by DCe also have very small average clustering coefficients and high average shortest path length, consistent with those observed by our method. These results supported our speculation that the tree-like structure of DCEN might be caused by the intrinsic characteristics of coexpression networks. Here, we mainly focused on the observed tree structure of DCEN and would uncover its corresponding biological significance and functional interpretation. In the future, it will be interesting to systematically investigate additional topological properties and further compare DCEN structure with various types of networks^26^,^27^.

### Interpretations of differential coexpression networks

Biological networks have been comprehensively used to investigate the molecular mechanisms underlying a specific biological process. Therefore, we questioned whether DCEN could reveal the molecular mechanisms in response to the change of biological conditions. We performed functional enrichment analysis on the components of DCEN, and found that the significantly enriched functions are indeed associated with the experimental observations. As DCEN1 was deduced from the expression data in response to changes in glucose concentrations, the functions of DCEN1 components were related to several metabolic processes (Table S3). DCEN2 was derived from the gene expression profiles of a yeast strain with mutations on YOX1 and YHP1, which are important transcription factors in the regulation of the cell cycle. Accordingly, many components of DCEN2 were found to be cell cycle-related proteins (Table S4). These results imply that the differential coexpression network can help reveal the underlying mechanisms during the change of biological conditions.

**Figure 3:**
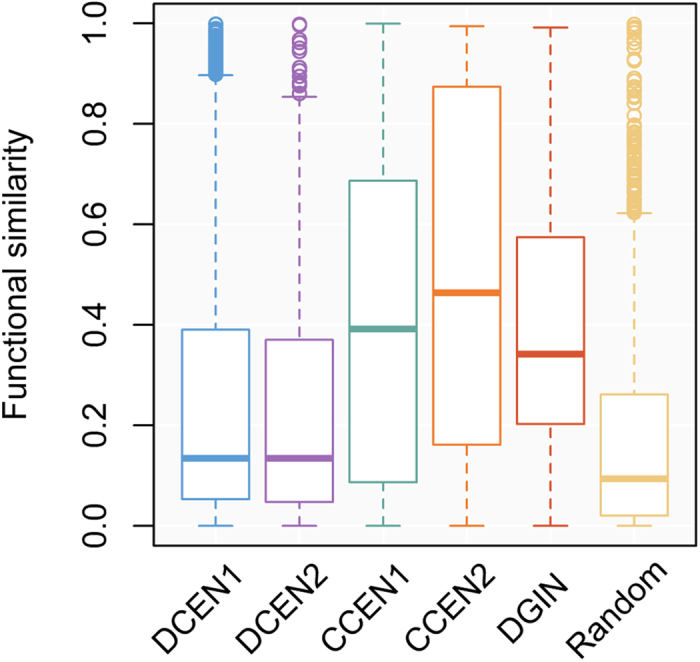
Distributions of functional similarities between genes. The functional similarities between genes were calculated based on GO semantic similarity. The CCEN and DGIN were used as positive controls because coexpression and genetic interaction were supported evidences in GO annotation. The random sets (Random) were negative controls. DCEN, differential coexpression network; CCEN, constitutive coexpression network; DGIN, differential genetic interaction network.

Next, we would like to interpret the biological significance of DCELs. Because many biological processes are achieved via protein-protein interactions (PPIs), we first examined these DCELs in a PPI database. After PPI searching in the BioGRID database^28^, surprisingly, only 10 links of DCEN1 and no links of DCEN2 were supported by PPI. This result reveals a limited relationship between DCELs and PPIs. We then examined whether two genes with differential coexpression are involved in similar biological processes. Gene Ontology (GO) semantic similarity was used to quantify the functional association of linked genes. Because coexpression patterns and genetic interactions have been used as evidence for gene function annotation by GO, the annotations with evidence codes of “inferred from expression pattern (IEP)” and “inferred from genetic interaction (IGI)” were discarded to avoid annotation bias. Unsurprisingly, links in CCEN tend to have highly functional relationships. However, most of the links in the DCEN and DGIN were not functional relationships (Fig. 3). Based on this observation, we presumed that the corresponding genes of a differential co-expressed link might not directly or indirectly interact in the same biological pathway, but contribute to different biological processes. This assumption is similar to the functional interpretations of genetic interactions in previous studies^28^,^29^. Genetic interactions can typically be interpreted by the between-pathway mode, in which the genetic interaction of bride genes operate in two pathways, or the within-pathway mode, in which the genetic interaction occurs between proteins within a single pathway^29^,^30^, and a high proportion of genetic interactions are associated with the between-pathway mode^31^.

To address the between-pathway characteristics, we proposed a computational procedure to construct the relationships within and between pathways. The components of DCENs were classified into functional clusters, which may be considered as specific biological processes or pathways, and the connections within and between clusters were assessed. These functional clusters can assist us in interpreting the biological significance of a DCEN.

**Figure 4:**
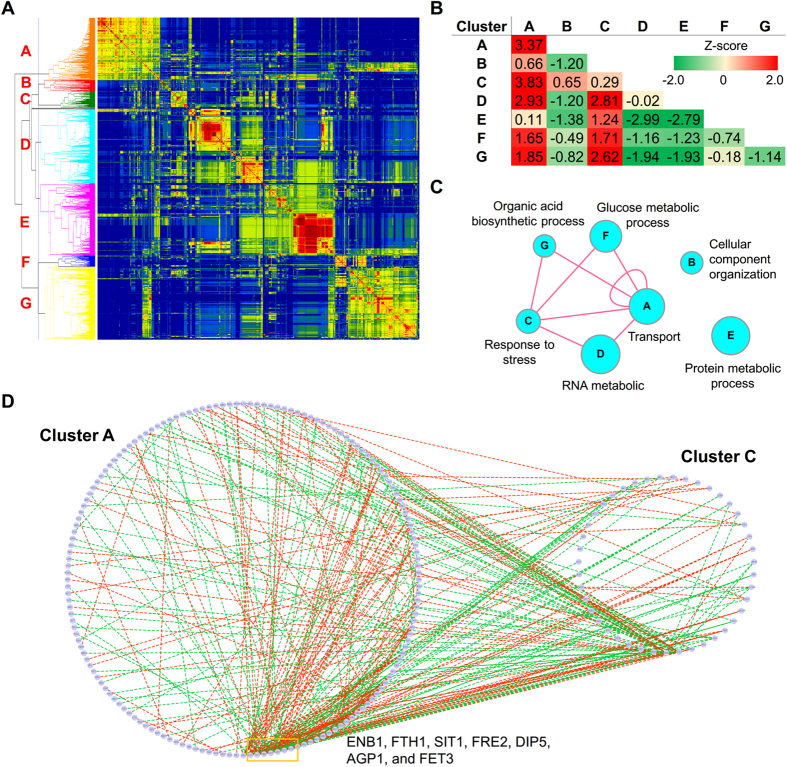
Functional clusters and their relationships of differential coexpression networks in response to glucose stress. (**A**) The heat map presents the functional similarity matrix among the components of a given DCEN. The red and blue colors represent high and low functional similarity, respectively. Based on the hierarchical tree, the components were divided into seven clusters (cluster **A**–**G**). (**B**) The table presents the z-transformed linking densities within- and between-clusters. (**C**) The interaction map of clusters was generated according to (**B**). The circle node denotes the cluster, and the edge represents the z-transformed linking density of linked clusters that was equal to or greater than 1.5. (**D**) The subnetwork of the given DCEN contains the interactions between cluster A and C. The circle node denotes the genes and the edge indicates the linked genes are differentially coexpressed. The red and green edges represent the positive and negative differential coexpression, respectively. Nodes in the given clusters are not shown if no edge linked to them.

In DCEN1, 1,269 components contained GO annotations and were used to generate a functional similarity profile. After hierarchical clustering, the components of DCEN1 were classified into seven clusters (Fig. 4A), and all clusters were significantly enriched for particular biological processes (Table S5). Based on these clusters, 2,300 and 510 DCELs belonged to between- and within-cluster types, respectively, and this ratio was significantly higher than that expected by chance (*P*-value = 0.0017). Then, the cluster relationships were examined, and we subsequently observed that genes in cluster A not only had high intra-interactions, but also strongly interacted with genes in other clusters, especially cluster C (Fig. 4B,C). Additionally, cluster C tended to interact with other clusters. Genes in cluster C were involved in the response to stress, which is consistent with previous studies that a change in the carbon source causes dramatic stress to yeast^22^,^32^. However, we were interested in the interaction between cluster A and C. Most genes in cluster A were related to molecular transport processes, and a subset of genes in cluster A connected with most genes in cluster C (Fig. 4D). We found these genes with high connections, including ENB1, FTH1, SIT1, FRE2 and FET3, are involved in the iron uptake pathway^33^,^34^,^35^. This observation may imply that iron uptake is required in response to carbon stress. Indeed, previous studies have indicated that a change in the carbon source can simultaneously induce the stress response and iron uptake pathways^33^,^34^.

**Figure 5:**
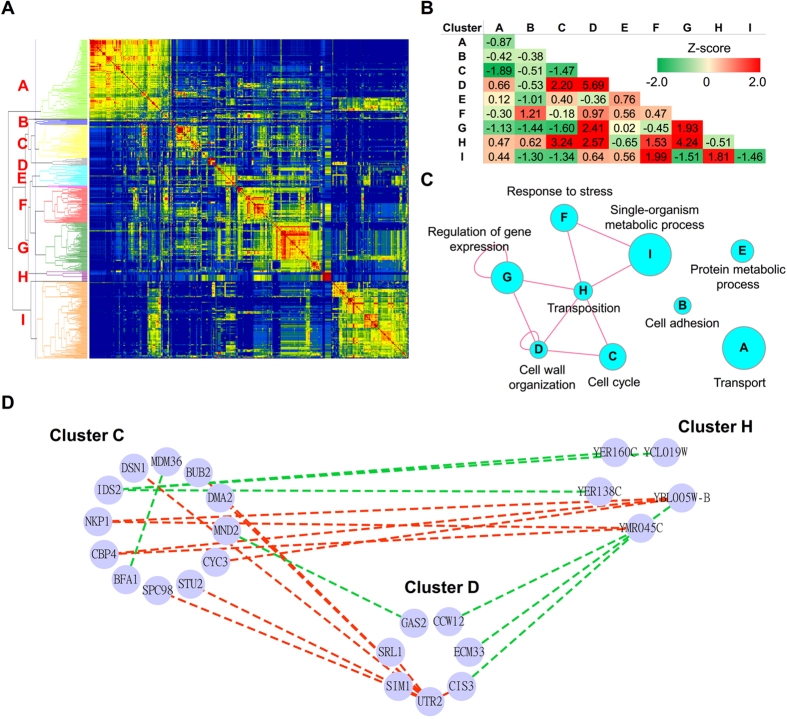
Functional clusters and their relationships of differential coexpression networks in response to deletions of YOX1 and YHP1. (**A**) The heat map presents the functional similarity matrix among components of a given DCEN. The red and blue colors represent high and low functional similarity, respectively. Based on the hierarchical tree, the components were divided into nine clusters (cluster A-I). (**B**) The table shows the z-transformed linking densities within- and between-clusters. (**C**) The interaction map of clusters was generated according to (**B**). The circle node denotes the cluster, and the edge represents the z-transformed linking density of linked clusters that was equal to or greater than 1.5. (**D**) The subnetwork of the given DCEN contains the interactions between cluster A and C. The circle node denotes the genes and the edge denotes the linked genes are differentially coexpressed. The red and green edges represent the positive and negative differential coexpression, respectively. The nodes in the given clusters are not shown if no edge linked to them.

In DCEN2, 518 components were classified into nine clusters (Fig. 5A), and a high proportion of DCELs belonged to the between-cluster mode (362 and 66 for between- and within-cluster modes, respectively, *P*-value = 0.0025). All clusters had significantly enriched biological processes (Table S6). Several clusters densely interacted with other clusters (Fig. 5B,C). Because the absence of YOX1 and YHP1 results in dysregulation of the cell cycle process, we focused on the interactions with cluster C, whose components were related to the cell cycle. Cluster C had significant connections with cluster D and H, whose components are related to cell wall organization and transposition, respectively. Previous studies indicated that YOX1 and YHP1 bind the promoters of genes involved in cell wall synthesis^36^, and many effectors of the cell wall integrity signaling pathway are active through the cell cycle^37^,^38^. Interestingly, the components of cluster H were all gag-pol fusion proteins that can regulate their own translation. However, the relationships between gag-pol proteins and cell cycle and cell wall organization are still unclear.

These results suggest that the differential coexpressed links typically span multiple pathways instead of occurring within a single pathway. Moreover, the linked pathways have clear interdependent functional relationships. One reason for the preference towards between-pathway types may be that these mechanisms increase the efficiency in response to environmental or genetic changes, or provide synergistic effects.

### Inferring differential activation of transcription factors

**Figure 6:**
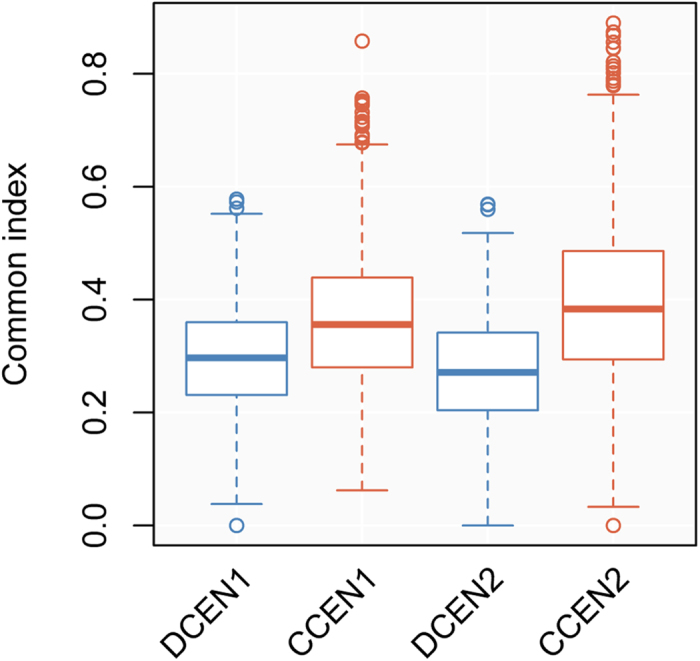
The distribution of common transcription factors binding to differentially or constitutively coexpressed genes. The common index is the arithmetic mean of the Jaccard and overlap coefficients. Suppose that we are given two gene lists that are bound by the set of transcription factors A and B, respectively. The Jaccard coefficient is defined as |A ∩ B| / | A ∪ B| and the overlap coefficient is defined as |A ∩ B| / max(|A|, |B|). DCEN, differential coexpression network; CCEN, constitutive coexpression network.

Finally, we would like to determine what mechanisms cause the differential coexpression phenomena. Transcription factors (TFs) are one of the major regulators in transcriptional control of gene expressions. TFs may coordinate by forming a complex, compete for promoter occupancy, or play antagonistic regulatory roles^39^. First, we examined the relationship between TFs and DCELs. Counting the number of common TFs between any pair of genes showed that CCELs tend to be regulated by more common TFs (Fig. 6), but DCELs do not. This suggests that gene pairs with more common regulators are robust to perturbations and tend to be coexpressed across various conditions, whereas gene pairs with less common regulators easily reveal differential coexpression when one of regulators are dysfunctional. Based on this observation, we presumed that the activated or deactivated transcription factors induced by environmental stress or genetic change could be identified from DCENs. A similar idea appeared in another study^40^.

We have taken a computational approach to infer differential activation of TFs inferred from DCENs. Our approach is based on the assumption that a TF is likely to be activated or deactivated across two conditions if significantly more differential coexpressed genes bound by this TF are observed than expected by chance. A total of 31 and 24 TFs revealing differential activation (*P*-value < 0.05) were identified from DCEN1 and DCEN2, respectively (Tables 2 and 3). Interrogating the expression correlation among targets of each TF, we found that the distributions of most TFs differed significantly between two conditions (Figure S6 and Figure S7). This might be alternative evidence to demonstrate that these TFs gain or lose activity under different perturbations. Moreover, many TFs have been known to be related to processes in response to the corresponding stress, and we discuss this in detail in the following two paragraphs.

Some TFs derived from DCNE1 are activators or repressors in the carbon-source metabolic pathways (Table 2). For example, ADR1 regulates genes involved in utilization of non-fermentable carbon sources^41^ whereas CAT8 regulates gluconeogenic genes^42^. However, both ADR1 and CAT8 are known to synergistically act for strong derepression of target genes, such as ADH2 and ACS1^43^, which are also the components of DCEN1. YAP1 is a transcription activator involved in oxidative stress response and accumulates in the nucleus in response to carbon stress^44^. The function of MIG3 is still unclear, but a recent study showed that MIG3 may be related to the glucose-signaling network^45^. Additionally, several TFs are associated with metal cation uptake, which is consistent with the observation in the functional cluster analysis (Fig. 4C). AFT1 and AFT2 are the iron responsive transcriptional activators that regulate a series of genes involved in cell surface iron uptake (FET3, FRE1, FRE2, and FRE3), siderophore uptake (ARN1, FIT1, and FIT3), and iron transport across the vacuole membrane (FET5 and FTH1)^46^,^47^,^48^. Moreover, AFT1 can interact with other TFs to regulate transport of other metals. For example, AFT1 and MAC1 might corporately regulate transcription of CTR2 to mediate the mobilization of vacuolar copper stores in yeast^49^. Other TFs, such as HSF1, regulate genes in response to stress or heat shock^50^.

A third of the differential activation of TFs inferred from DCEN2 is yeast cell-cycle transcription factors (Table 3). Among these TFs, YOX1 and YHP1 were respectively ranked as the top 1 and 5 TFs according to their *P*-values. This result may also demonstrate the ability of our approach for inferring differential activation of TFs. Moreover, MCM1, SWI4, SWI6, MBP1, FKH1, and FKH2 are involved in activating gene expression during the cell cycle in yeast. YOX1 and YHP1 act in concert with MCM1 to confer M/G1-specificity to the early cell cycle box (ECB) elements^23^. SBF, a heterodimer of SWI4 and SWI6, and MBF, a heterodimer of MBP1 and SWI6, are sequence-specific transcription factors that activate gene expression during the G1/S transition of the cell cycle in yeast. SBF binds to the so-called SCB (Swi4,6-dependent cell cycle box) promoter elements found upstream of the cyclin genes and cell wall biosynthetic genes, while MBF binds to a distinct element called the MCB (MluI cell cycle box) found mostly upstream of DNA replication and repair genes^51^,^52^,^53^,^54^. FKH1 and FKH2, forkhead transcription factors, assemble into ternary complexes with MCM1 to control transcription required for M-phase^55^. However, YOX1 competes with FKH2 for binding to MCM1 through protein-protein interactions at promoters of a subset of MCM1-regulated genes^56^.

Identifying the regulators that are relevant or even causative to a phenotypic change is a challenging goal. This problem cannot be solved using traditional differential expression analysis because a causal regulator is not necessarily differentially expressed. However, the causal regulators might be captured from DCENs. For example, a previous study that used a differential wiring analysis of expression data succeeded in identifying the gene containing the causal mutation^57^. Our method successfully identified several transcription factors, which have been demonstrated to be associated with corresponding phenotypes. We also found that many TFs are not the components of DCENs (Tables 2 and 3). This indicates that the expression of these TFs is not dramatically perturbed across the conditions, but the activity of these TFs might still be influenced. Interestingly, the functions in which TFs are involved are consistent with the results of functional clustering analysis (Figs 4C and 5C). In brief, we demonstrated the ability of DCENs in understanding the dynamics and regulatory mechanisms of cellular systems.

## Conclusions

In this work, we uncovered the unique structural characteristics and biological significance of differential coexpression networks (DCENs). DCENs resemble a tree-like network due to the intrinsic properties of coexpression networks. Although they possess the scale-free property, DCENs have a lower average clustering coefficient and higher average shortest path length in contrast to other biological networks. Furthermore, we proposed new approaches to the interpretation of DCEN. Our analysis found that differentially coexpressed genes tend to participate in different pathways. Pathways linked by differentially coexpressed genes may play complementary functions or have synergistic effects. We integrated transcription factor information with DCEN to reveal the regulatory mechanisms inducing differential coexpression. In brief, these results demonstrate that DCENs provide insights into the gene regulatory dynamics in response to various stresses. Additionally, the computational procedures proposed in this study can be applied to other coexpression networks, such as disease mechanism studies.

## Methods

### Gene expression data pre-processing

The raw expression data were obtained from Gene Expression Omnibus (GEO)^21^ and processed using the following steps. First, the gene expression data were normalized using a quantile normalization method to ensure a similar empirical distribution of each array. Second, probes with more than 20% of missing time points for a condition were discarded. Third, because the missing data were not allowed in the correlation analysis, the remaining missing data were imputed using the *k*-nearest neighbors (KNN) technique. In this step, we used the KNN algorithm implemented in the R package “impute” with *k* = 10 to impute the missing values. Fourth, the probes without annotation were removed from further analysis, and if the probes were derived from the same gene, the expression intensity of this gene was represented by the average intensity of these probes. Finally, the genes with low variance across all time points were ignored for further analysis. Because these genes were constitutively expressed during all time points and were not perturbed under any condition, they might be non-informative for a coexpression network. We calculated the standard deviation of each gene to represent the perturbation of this gene under a condition, and if the standard deviation of a gene in both conditions were not in the top 25% of all genes, the gene was removed.

### Construction of differential coexpression network

We modified the method proposed by Wally *et al.*^20^ to identify differentially coexpressed links (DCELs). The difference of coexpression of gene *u* and *v* between two conditions *a* and *b* was quantified by the following formula,


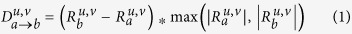


where 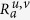 is the correlation between gene *u* and *v* at condition *a*. The latter term of this equation (i.e., 
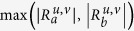
) was used to distinguish the importance when the values of the former term were identical. For example, the correlation of two pairs of genes between two conditions ranges from 1.0 to 0.5 and from 0.5 to 0.0. Although the correlation difference of these pairs is −0.5, the former case might be more informative than the latter. Under the definition of equation (1), positive aggregated scores mark the cases in which the correlation in condition *b* is higher than in condition *a*; we refer to this situation as a “positively differentially coexpressed link”. Negative aggregated scores indicate a lower correlation between the pair in condition *b*, referred to as a “negatively differentially coexpressed link”. We calculated the correlation between genes using the Spearman correlation coefficient.

Significant differences were evaluated using permutation testing with different resample schemes chosen according to the dependency of the two samples. After 100,000 randomizations, empirical *P*-values were computed as the proportion of the difference observed in the permuted data set that was equal to or greater than that observed in the original data. Then, empirical *P*-values were adjusted using the Benjamini-Hochberg (BH) procedure to account for multiple hypothesis testing. The gene pairs with *P*-values < 10^−4^ were considered as DCELs, and then these DCELs were assembled into a DCEN.

### Differential genetic interaction network

To compare the differences between various differential networks, we obtained a differential genetic interaction network (DGIN), derived from the comparison of two genetic interaction networks with and without perturbation by the DNA-damaging agent methyl methanesulfonate (MMS)^8^. This study identified 78,841 genetic interactions and 873 differential genetic interactions with *P*-value < 0.001 covering 318 genes. A total of 379 interactions were “negatively differential”, which indicates DNA damage-induced lethality or sickness, whereas 494 were “positively differential”, which indicates inducible epistasis or suppression.

### Topological properties

To investigate the structural properties of differential networks, the following network topologies were quantified.

1. The degree *k*_*i*_ of a vertex indicates the number of other vertices connected with this vertex. The average degree *< k >* is the overall mean *k*_*i*_ of network vertices. The degree distribution (*P*(*k*)) is defined as the fraction of vertices in the network having degree *k*. Most of the real networks display a power law shaped degree distribution *P*(*k*) ~ *k*^*-*γ^, where γ is a constant usually between 1 and 3. We estimated the exponent γ of each network using the Python package powerlaw^58^.
2. The shortest path length *d*_*i*_ of two vertices represents the number of edges along the shortest path connecting them. The average shortest path length (*L*) and diameter (*D*) are, respectively, defined as the mean and maximum *d*_*i*_ across all vertex pairs in the network.
3. The clustering coefficient *c*_*i*_ of a vertex is defined as the ratio between the number of connections existing between its neighbors and the maximal number of edges that can exist between them. The average clustering coefficient (*C*) and the degree-dependent clustering coefficient (*C*(*k*)) are the mean *c*_*i*_ across all network vertices and the overall vertices with degree *k*, respectively.

### Gene Ontology enrichment analysis

The GO terms were obtained from the GO website (http://www.geneontology.org/), and the GO annotations for all yeast genes were downloaded from NCBI Entrez Gene. The enrichment analysis was performed using a hypergeometric test. Suppose that we are given a test set with *n* genes of which *k* genes belong to a certain GO term *g*, and a reference set with *N* genes of which *M* genes belong to *g*. The probability of *g* can be calculated according to the following formula.


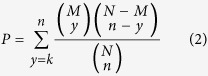


Then, the adjustment for multiple comparisons was undertaken using the BH procedure. We only considered the “Biological Process” ontology in this work.

### Functional semantic similarity between genes

The functional similarity between genes was measured by the semantic similarity between sets of GO terms with which they were annotated. We applied the method proposed by Schlicker *et al.*^59^ to quantify the functional similarity. Schlicker’s measurement method combines Resnik’s method, which uses the concept of “information content” to define a semantic similarity^60^, and Lin’s method, which defines the similarity between two terms as the ratio of the commonality of the terms and the information needed to fully describe the terms^61^.

The first step in the comparison of two genes is the pairwise comparison of their GO mappings. Considering two genes A and B annotated with the sets of GO terms with sizes *N* and *M*, respectively, a similarity matrix *S* is calculated. This matrix contains all pairwise semantic similarity values between GO terms of gene A and GO terms of gene B. For the *i-*th GO term gene A, 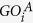, and the *j*-th GO term of gene B, 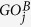, the semantic similarity score *s*_*i,j*_ of Schlicker’s method is defined as:


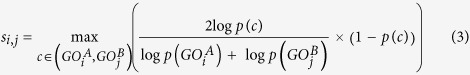


where *c* is the set of common ancestors of term 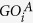 and 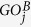, and *p*(*c*) denotes the probability of the term *c* that is equal to its frequency in the annotations. The functional similarity of gene A (*G*^*A*^) and B (*G*^*B*^), *funSim*, is calculated using the best-match average (BMA) of the matrix *S*:


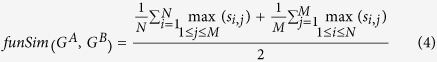


### Identification of functional clusters and densities among clusters

Genes in DCEN were clustered based on their functional similarity profiles. In brief, the functional similarity between any given pair of genes was computed using GO semantic similarity as mentioned previously and a functional similarity matrix was formed. Hierarchical agglomerative average-linkage clustering with the Pearson correlation coefficient as the distance metric was applied to the functional similarity matrix. Generalized Association Plots (GAP)^62^ were used to perform hierarchical clustering analysis and to view the results. The distance thresholds were determined based on the visual inspection of the heatmap and hierarchical trees.

The connectivity densities of DCELs (*D*_*i,j*_) within and between clusters were calculated based on following formula:


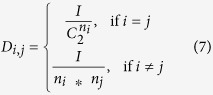


where *I* is the number of DCELs within a single cluster of genes (if *i* = *j*) or connecting two clusters (if *i* ≠ *j*), and *n*_*i*_ is the number of genes in the cluster *i*. Because the density is difficult to interpret, the density was transformed into a z-score. To calculate the z-score, the background distribution of connectivity density was estimated by randomizing the gene-cluster association and re-calculating the connectivity density. After 100,000 randomizations, the mean (*μ*) and standard deviation (*ρ*) of the background distribution were obtained, and the z-score of density between cluster *i* and *j* (*z*_*i,j*_) is given by the formula:


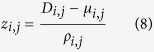


As the z-score > 1.5, it indicates that the connections within a given cluster or between the given clusters is significantly greater than that expected by chance.

We also used the same randomization procedure to estimate the significance of the ratio of between-cluster to within-cluster DCELs. The empirical *P*-value was calculated by counting the number of permutations in which the ratio of between-cluster to within-cluster DCELs was greater than or equal to the observed ratio, and then divided that number by the total number of permutations.

### Identification of differential activation of transcriptional factors

To investigate the regulators of differentially coexpressed genes, the relationships between transcriptional factors (TFs) and regulated genes in budding yeast were obtained from YEASTRACT^63^. A total of 191,902 TF-gene regulations were available, including 315 transcriptional factors and 5,963 genes.

We proposed a computational approach to identify differential activation of transcription factors from DCENs. For each TF, the number of DCELs in which both genes were regulated by the given TF was counted. Next, the background distribution of the co-occurrence count of each TF was estimated by a randomization procedure as follows. For each component of a DCEN, the number of TFs regulating the given gene was counted, and a gene that was regulated by the same or a similar number of TFs was randomly selected from all yeast genes and used to replace the given gene. Once all components of a DCEN were randomized, the number of co-occurrence on both genes for all TFs was counted. This randomization step was repeated 100,000 times. The empirical *P*-value of a TF was calculated by counting the fraction of permutations in which the random co-occurrence count was higher than or equal to the observed value, and then corrected using the BH method to control the false discovery rate. The TFs with *P*-value < 0.05 were considered as differentially activated.

## Additional Information

**How to cite this article**: Hsu, C.-L. *et al.* Functional Analysis and Characterization of Differential Coexpression Networks. *Sci. Rep.* **5**, 13295; doi: 10.1038/srep13295 (2015).

## Supplementary Material

Supplementary Information

## Figures and Tables

**Table 1:** Characteristics of differential networks.

Quantity	DCEN 1: 0.2 g/l glucose → 2.0 g/l glucose	DCEN 2: Wild-type → deletions of YOX1 & YHP1	DGIN: No treatment → MMS treatment
Nodes	1,380 (1,425)	300 (611)	318
Edges	3,387 (3,411)	341 (559)	873
Positive differential	1,779 (1,795)	184 (284)	494
Negative differential	1,608 (1,616)	157 (275)	379
< *k *>	4.91	2.27	5.49
Max. *k*	208	29	54
γ	2.43	2.44	3.4
*C*	0.0025	0	0.069
*D*	11	26	7
*L*	4.31	8.50	3.23

The numbers in parentheses are the corresponding quantity values of the whole network. *< k >*, average degree; Max. *k*, maximum degree; *C*, average clustering coefficient; γ, exponent of degree distribution; *D*, diameter of a network; *L*, average shortest path length.

**Table 2:** Differential activation of transcription factors induced by glucose stress.

Transcription factor	# co-occurrence	P-value	Transcription factor	# co-occurrence	P-value
PDR1	641	<0.00001	MAC1	106	0.00539
SFP1	2624	<0.00001	AFT2	100	0.00675
AFT1	566	0.00007	OPI1*	73	0.00859
STB1	73	0.0001	UME6	215	0.00896
PDR3	384	0.00011	GZF3	45	0.01054
SWI5*	875	0.0003	MIG3*	475	0.01172
ADR1*	255	0.00049	CAT8*	24	0.01176
HAP3	47	0.00078	GCN4	1405	0.01394
MSN4	1034	0.00086	YAP1	1309	0.02051
ZAP1	548	0.00182	RIM101	190	0.02827
RTG3	180	0.00205	RME1	54	0.03382
DAL81	62	0.00308	HSF1	614	0.04566
ROX1*	248	0.00316	SMP1	26	0.04606
SOK2*	1033	0.00342	OAF1*	198	0.04678
ACE2*	2638	0.00413	GAT1	18	0.04729
MET4	582	0.00454			

^*^TFs are also the components of differential coexpression network.

**Table 3:** Differential activation of transcription factors induced by deletions of YOX1 and YHP1.

Transcription factor	# co-occurrence	P-value	Transcription factor	# co-occurrence	P-value
YOX1	86	0.00001	SWI4	43	0.0128
STE12	288	0.00011	ROX1	33	0.01608
RIM101	39	0.00015	FKH1*	25	0.01667
CIN5	138	0.00019	FKH2*	17	0.01867
SPT23	98	0.00077	SOK2*	128	0.02385
YHP1*	64	0.00145	GCN4	195	0.02526
IXR1*	82	0.00179	KAR4*	25	0.03046
TEC1	262	0.00346	INO2	12	0.04265
SWI6*	14	0.00498	SUM1	15	0.04679
MCM1	82	0.00856	FLO8	18	0.04835
PHD1	26	0.01135	SPT3	42	0.04891
CRZ1	15	0.0116	MBP1	18	0.04923

^*^TFs are also the components of differential coexpression network.
