# Supplementary material for: Functional Analysis and Characterization of Differential Coexpression Networks
Source: Sci Rep. 2015 Aug 18;5:13295. doi: 10.1038/srep13295 (PMC4539605; doi:10.1038/srep13295)
Supplement: Supplementary Information [file srep13295-s1.pdf]

Supplementary Information

Functional Analysis and Characterization of Differential Coexpression Networks

Chia-Lang Hsu[1], Hsueh-Fen Juan[1,2,3,*], and Hsuan-Cheng Huang[4,*]

[1]Department of Life Science, [2]Graduate Institute of Biomedical Electronics and Bioinformatics, [3]Institute of Molecular and Cellular Biology, National Taiwan University, Taipei 10617, Taiwan, [4]Institute of Biomedical Informatics, Center of Systems and Synthetic Biology, National Yang-Ming University, Taipei 11221, Taiwan

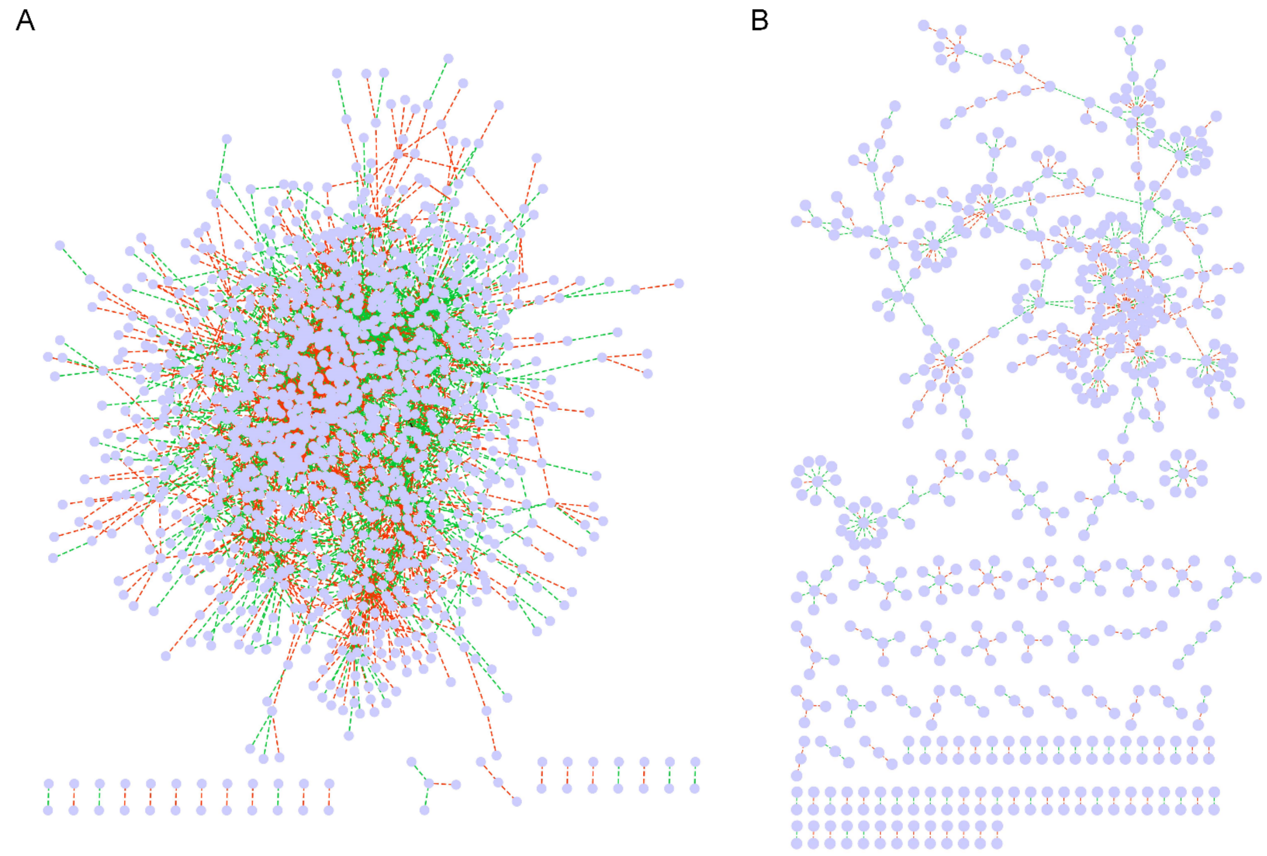

Figure S1. Graphic view of differential coexpression networks

The differential coexpression networks induced by glucose stress (A) and deletions of YOX1 and YHP1 (B). Nodes denote the genes and edges denote the differential coexpression links. The red and green edges represent the positively and negatively differential coexpression, respectively.

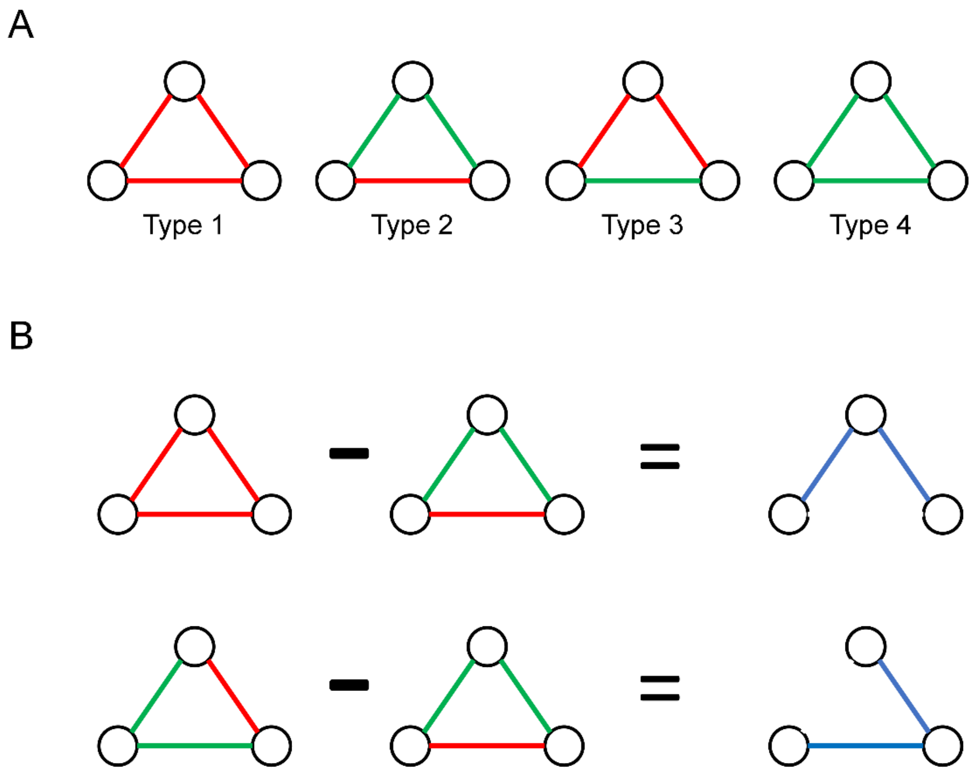

Figure S2. Illustration of triads in coexpression networks

There are four possible types of triads according to the combinatorial patterns of the three interconnected signed links (A). However, only type 1 and 2 triads can be observed in coexpression network because of the transmission characteristics of correlation. Because coexpression networks contain this specific property, no complete differential coexpression triad can be obtained (B). The red and green links denote the positive and negative correlation pairs. The blue link represents the significantly differential coexpressed link.

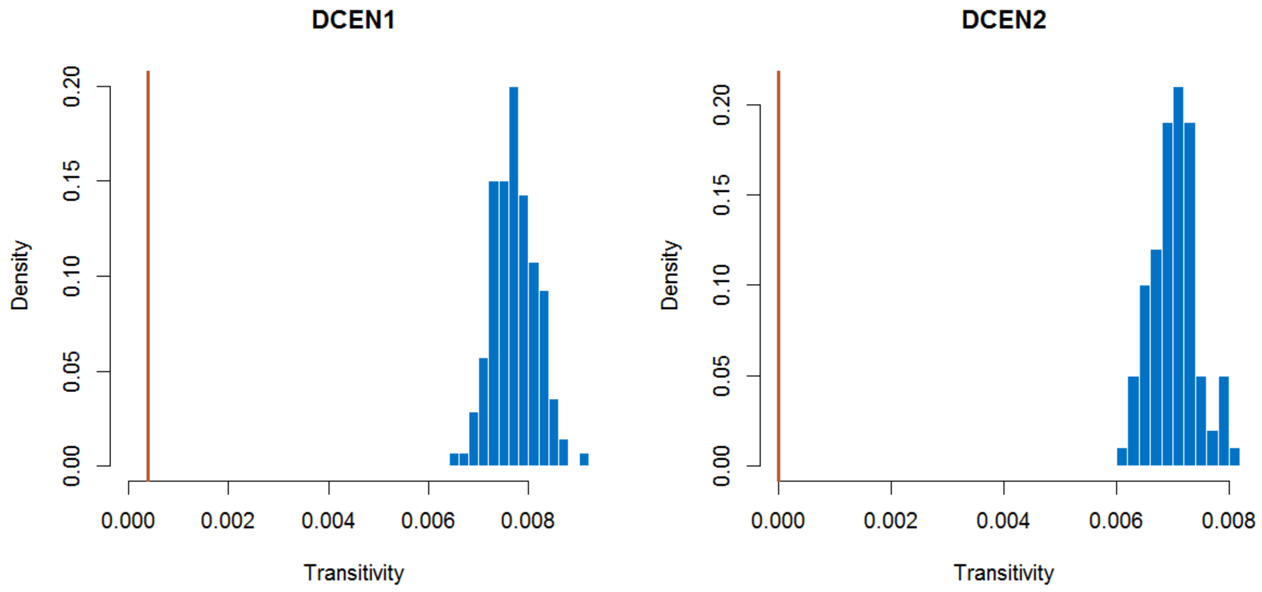

Figure S3. Distributions of transitivity in differential coexpression networks

The transitivity of a graph T is defined as $T = \frac{3\lambda}{\tau}$, where λ is the number of triads and τ is the number of triples. A triad is a complete subgraph with exact three nodes, and a triple is a subgraph with three nodes and two edge. The blue histogram represents the random distribution of transitivity of 100,000 randomized differential coexpression networks (DCENs) and Red line denotes the observed value of the given DCEN. A randomized DCEN was derived from gene expression data which relations between genes and expression profiles in a condition are randomized.

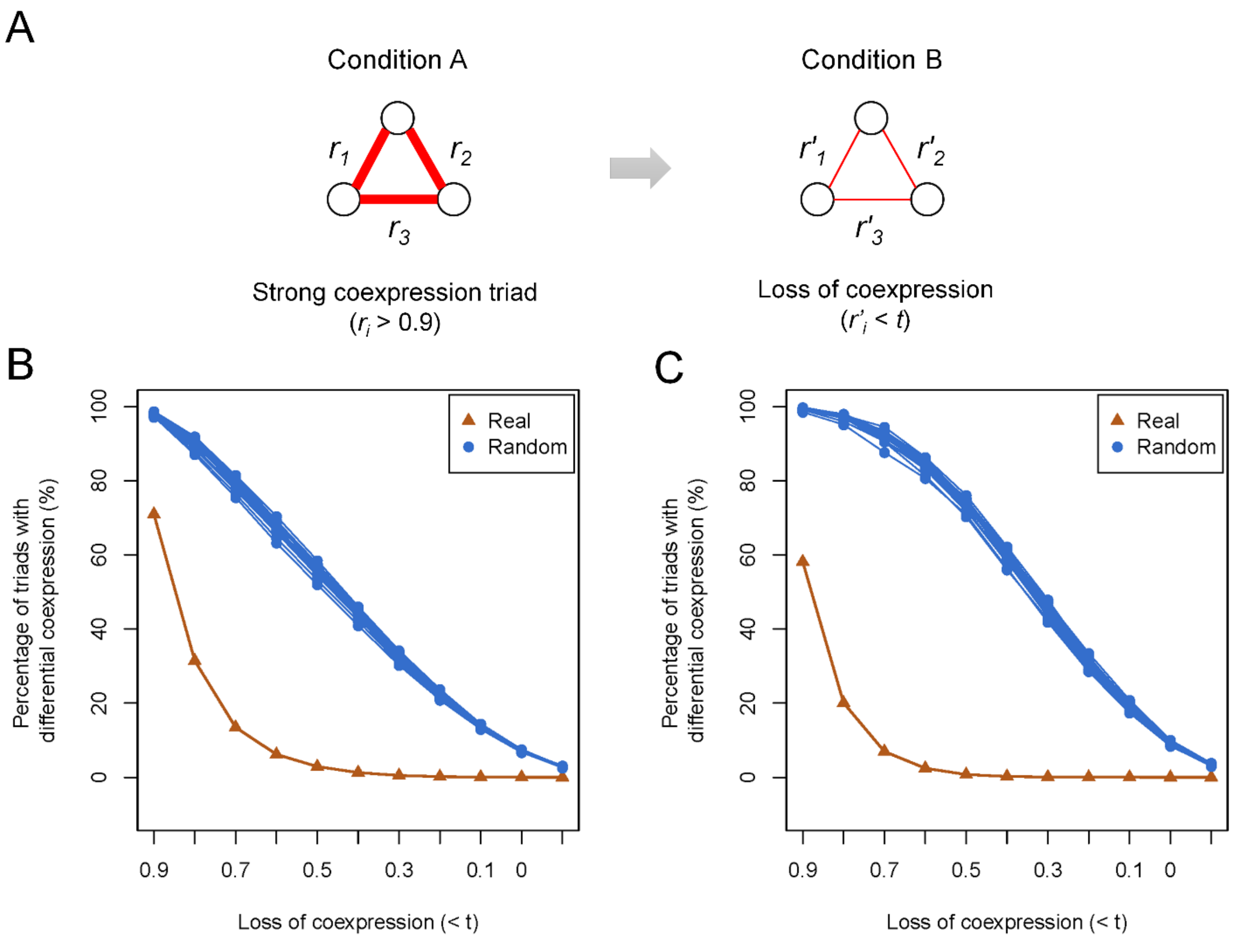

Figure S4. Differential coexpression analysis of strong coexpression triads.

A strong coexpression triad is defined as a triad in which expression profiles among genes are highly correlated in one condition (Spearman correlation coefficient > 0.9), and then we examined if these strong triads lose coexpression in another condition (A). The percentages of strong triads with loss of coexpression derived from dataset 1 and 2 are illustrated in (B) and (C), respectively. Red and blue lines represent the results from real and randomized gene expression data, respectively.

A

	Nodes	Edges	<*k*>	Max *k*	γ	*C*	*D*	*L*
**Dataset1**	1885 (1887)	8704 (8705)	9.23	215	3.0	0.014	9	3.56
**Dataset2**	712 (1086)	786 (1044)	2.21	11	3.7	0.006	28	10.07

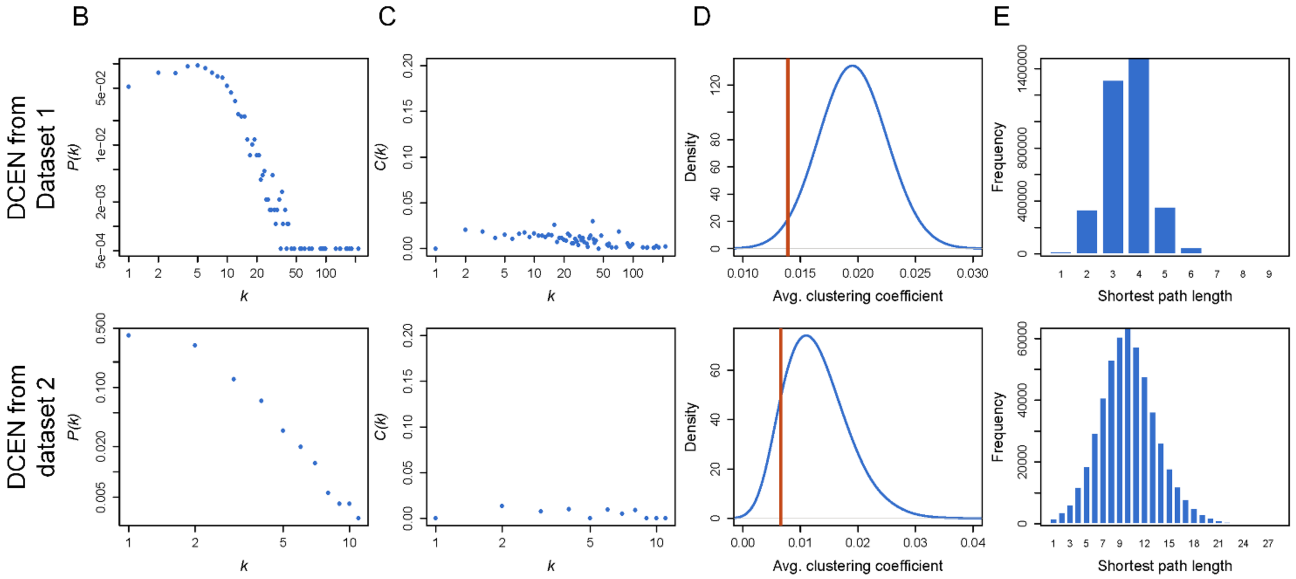

Figure S5. Topological properties of differential coexpression networks constructed by DCe
The method DCe, implemented in R package DCGL, was applied to the two gene expression datasets, respectively. The used parameters are as follows: link filtering method is “qth”; cutoff used for link filtering is 0.1; correlation coefficient is computed by spearman method; and the cutoff of q-value is 0.01. (A) Summary of topological properties. The numbers in parentheses are the corresponding quantity values of the whole network. <*k*>, average degree; Max. *k*, maximum degree; *C*, average clustering coefficient; γ, exponent of degree distribution; *D*, diameter of a network; *L*, average shortest path length. (B) Degree distributions of differential networks. *P(k)* is the number of genes with k links. (C) Clustering coefficient versus degree for differential networks. *C(k)* denotes the average clustering coefficient of all genes with k links. (D) Distributions of average clustering coefficient for random networks (blue line). The red line denotes the average clustering coefficient of the given differential networks. (E) Distributions of shortest path lengths for differential networks.

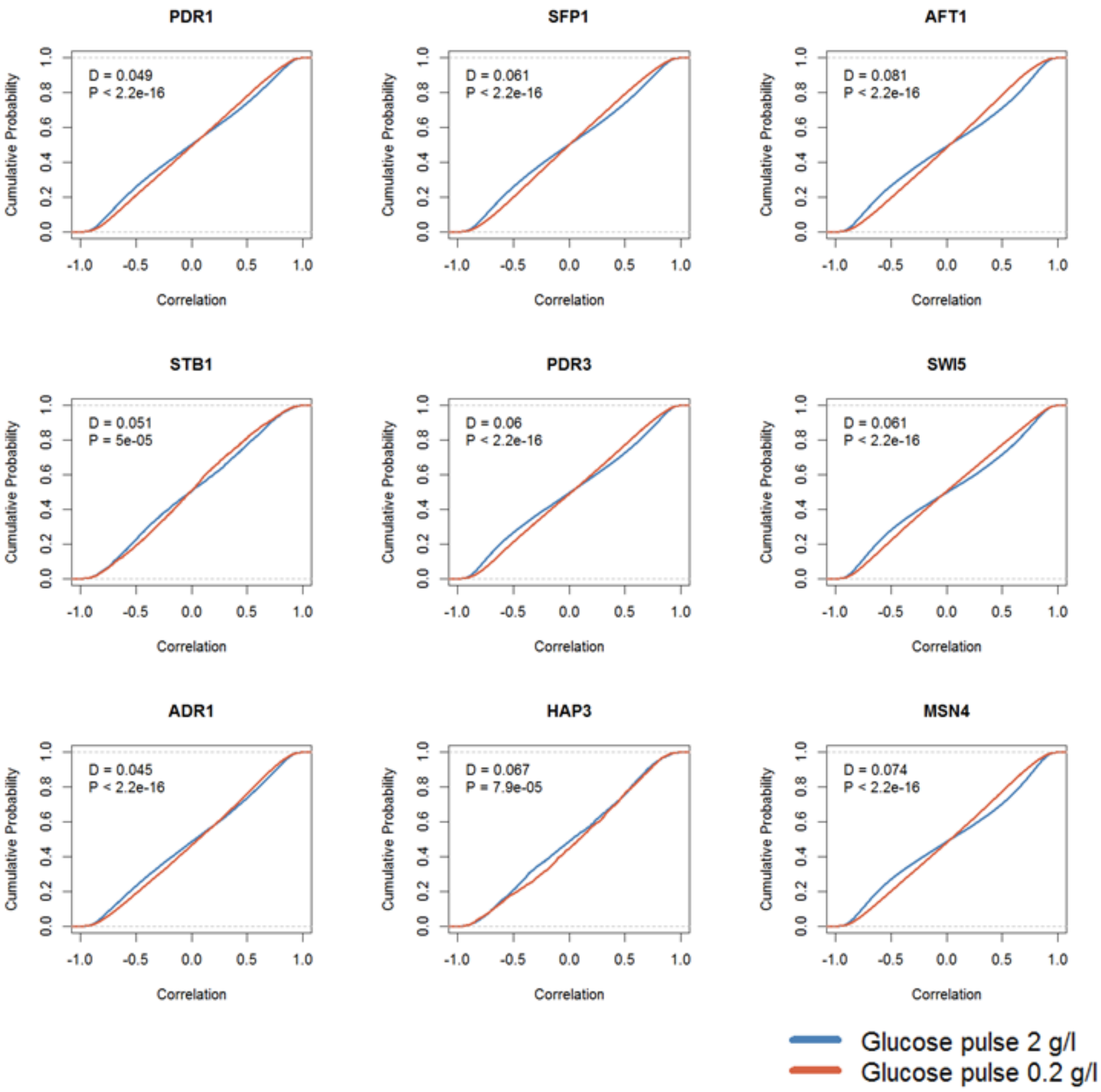

Figure S6. Cumulative distributions of expression correlation of TF co-targets under different glucose concentration

For each TF (Table2), the expression correlations of pair-wise combinations of the genes which are target by the give TF are presented. The D is the distance between two cumulative distributions and the P is the p-value of Kolmogorov–Smirnov test.

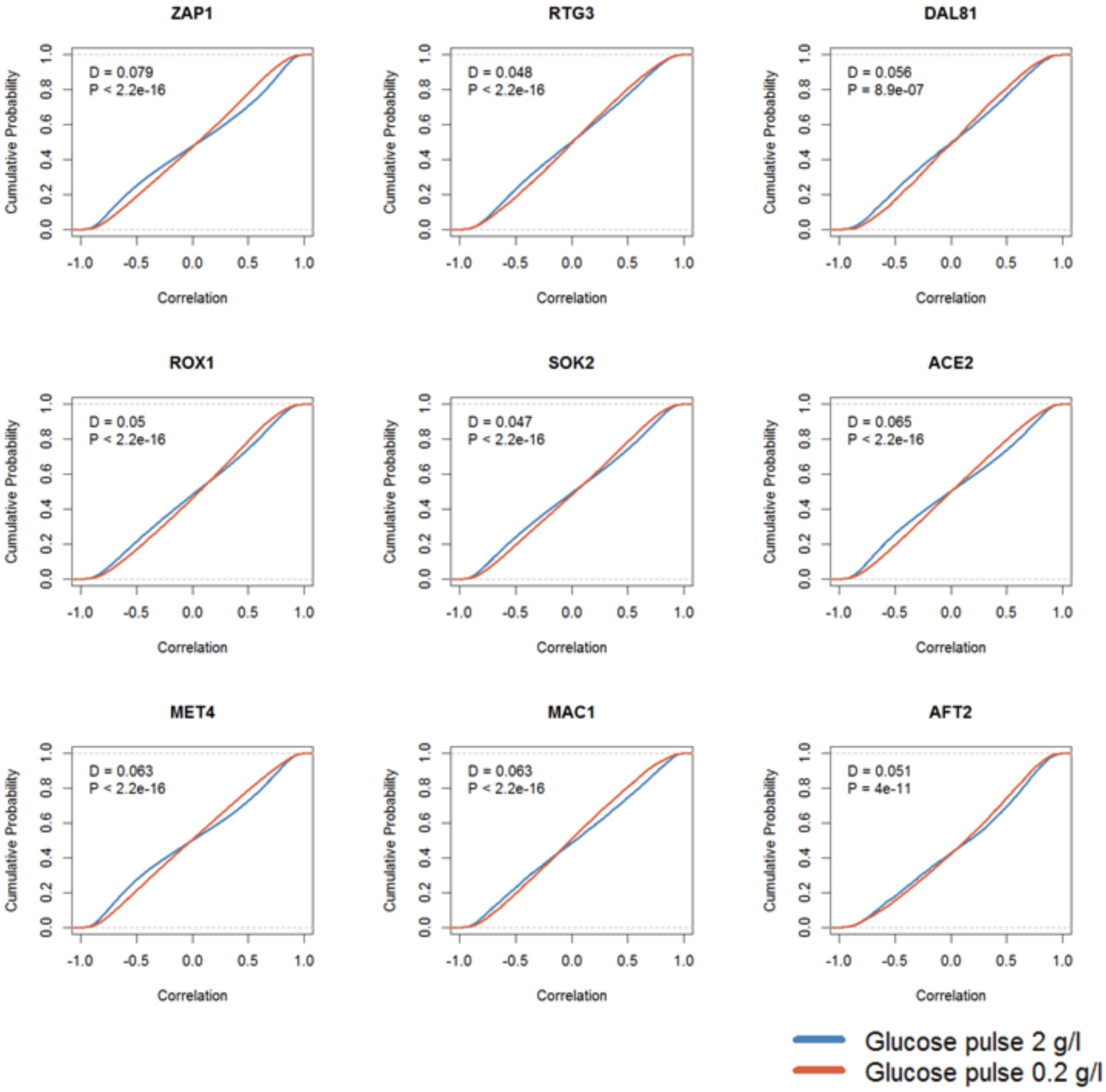

Figure S6. (cont.) Cumulative distributions of expression correlation of TF co-targets under different glucose concentration

For each TF (Table2), the expression correlations of pair-wise combinations of the genes which are target by the give TF are presented. The D is the distance between two cumulative distributions and the P is the p-value of Kolmogorov–Smirnov test.

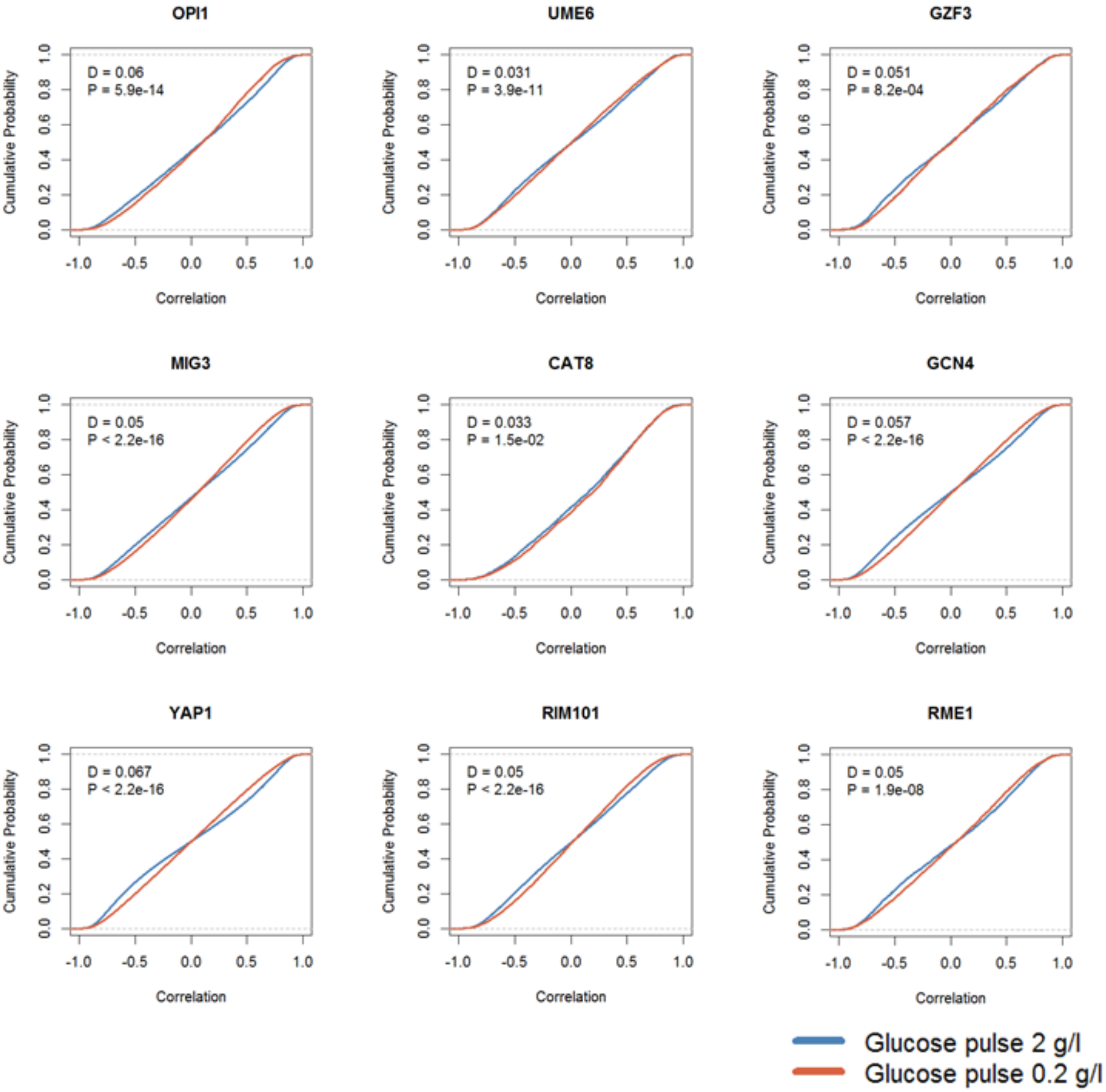

Figure S6. (cont.) Cumulative distributions of expression correlation of TF co-targets under different glucose concentration

For each TF (Table2), the expression correlations of pair-wise combinations of the genes which are target by the give TF are presented. The D is the distance between two cumulative distributions and the P is the p-value of Kolmogorov–Smirnov test.

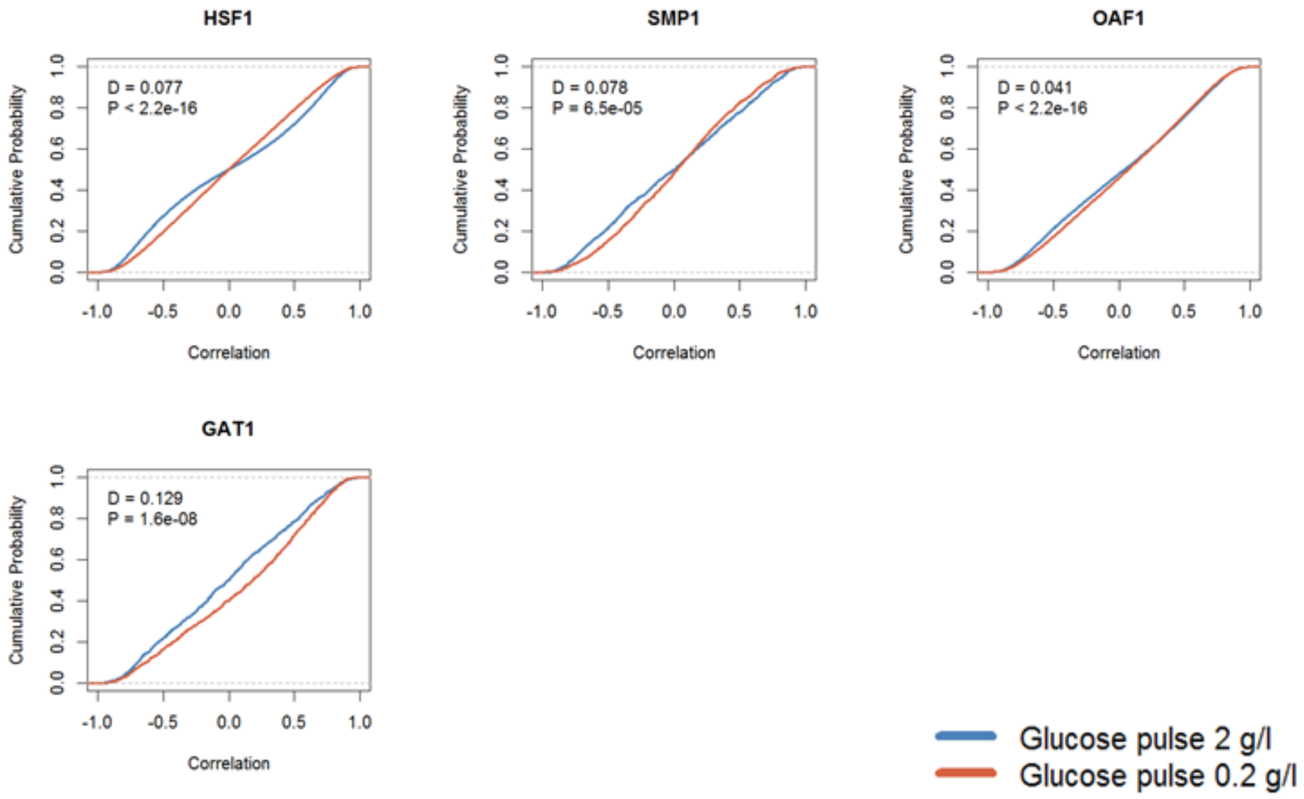

Figure S6. (cont.) Cumulative distributions of expression correlation of TF co-targets under different glucose concentration

For each TF (Table2), the expression correlations of pair-wise combinations of the genes which are target by the give TF are presented. The D is the distance between two cumulative distributions and the P is the p-value of Kolmogorov–Smirnov test.

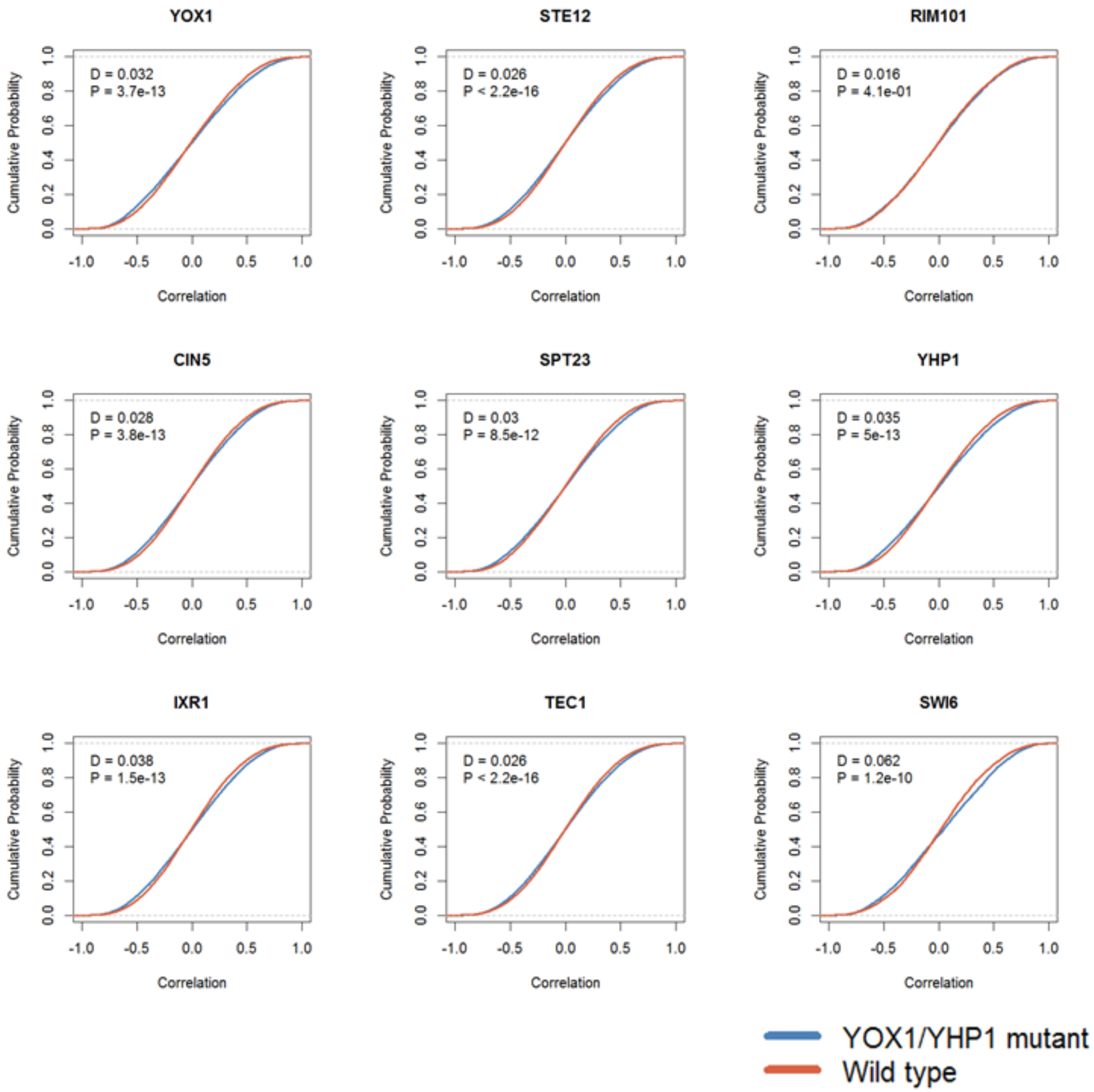

Figure S7. Cumulative distributions of expression correlation of TF co-targets in wide type and YOX1/YHP1 mutant

For each TF (Table 3), the expression correlations of pair-wise combinations of the genes which are target by the give TF are presented. The D is the distance between two cumulative distributions and the P is the *P*-value of Kolmogorov–Smirnov test.

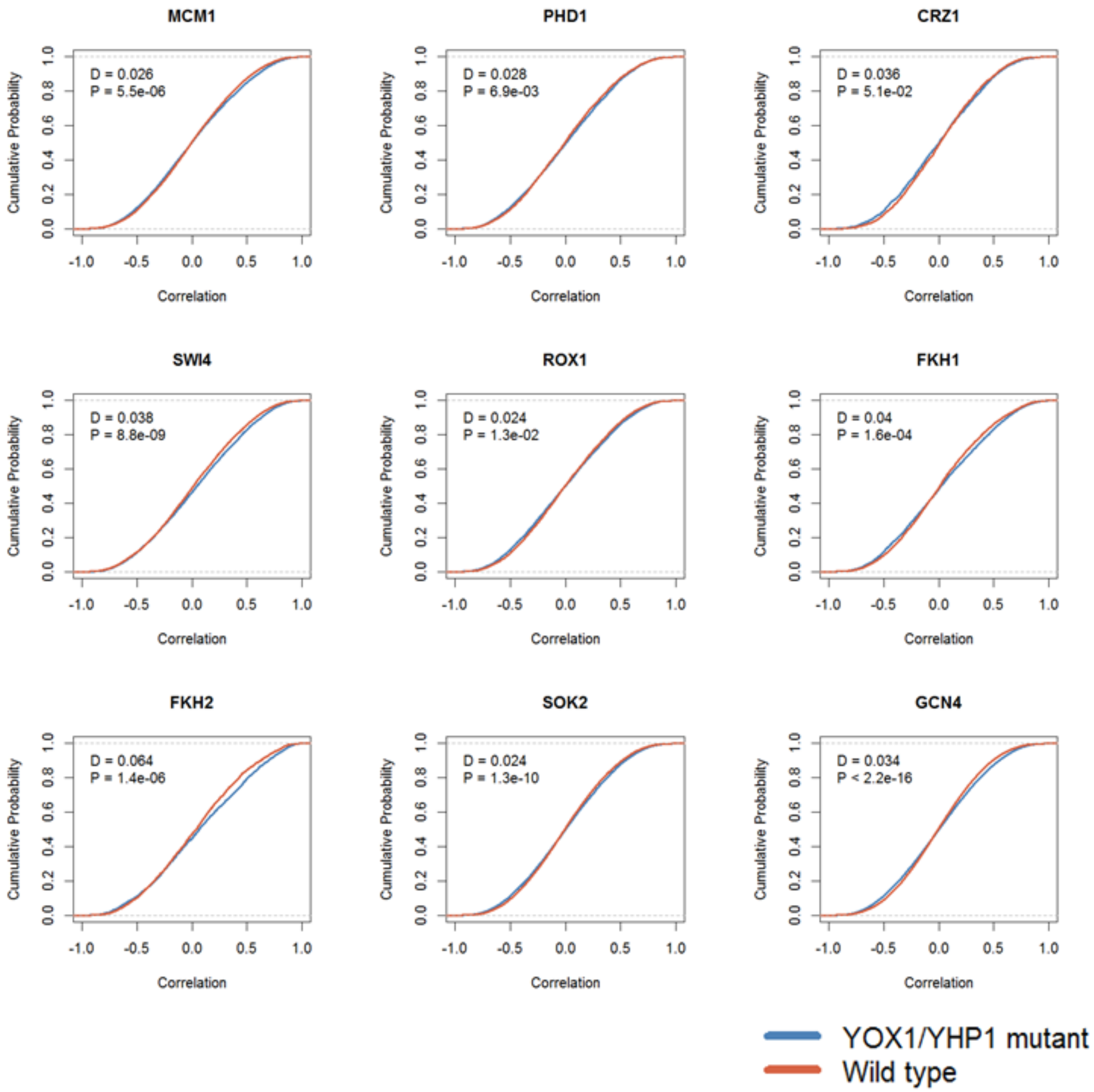

Figure S7. (cont.) Cumulative distributions of expression correlation of TF co-targets in wide type and YOX1/YHP1 mutant

For each TF (Table 3), the expression correlations of pair-wise combinations of the genes which are target by the give TF are presented. The D is the distance between two cumulative distributions and the P is the *P*-value of Kolmogorov–Smirnov test.

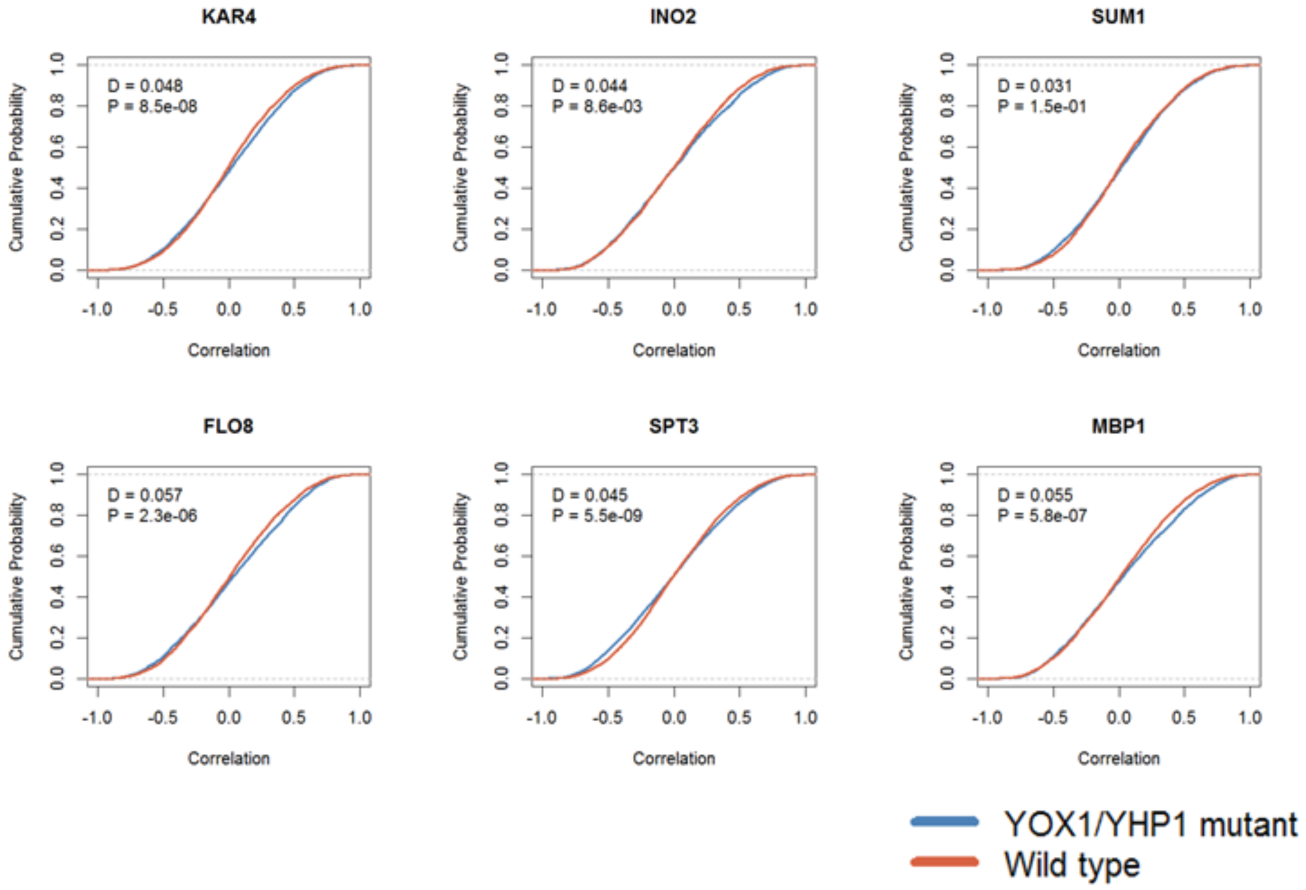

Figure S7. (cont.) Cumulative distributions of expression correlation of TF co-targets in wide type and YOX1/YHP1 mutant

For each TF (Table 3), the expression correlations of pair-wise combinations of the genes which are target by the give TF are presented. The D is the distance between two cumulative distributions and the P is the *P*-value of Kolmogorov–Smirnov test.
